# Knowing the enemy: ant behavior and control in a pediatric hospital of Buenos Aires

**DOI:** 10.1186/2193-1801-3-229

**Published:** 2014-05-06

**Authors:** Roxana Josens, Francisco J Sola, Nahuel Marchisio, María Agostina Di Renzo, Alina Giacometti

**Affiliations:** Grupo de Estudio de Insectos Sociales, IFIBYNE-CONICET, DBBE-Facultad de Ciencias Exactas y Naturales, Universidad de Buenos Aires Pabellón II, Ciudad Universitaria (C1428EHA), Buenos Aires, Argentina

**Keywords:** Ant, Chemical control, Baits, Food preferences, *Nylanderia fulva*

## Abstract

Ant control is difficult in systems even where a variety of control strategies and compounds are allowed; in sensitive places such as hospitals, where there are often restrictions on the methods and toxicants to be applied, the challenge is even greater. Here we report the methods and results of how we faced this challenge of controlling ants in a pediatric hospital using baits. Our strategy was based on identifying the species present and analyzing their behavior. On the one hand, we evaluated outdoors in the green areas of the hospital, the relative abundance of ant genera, their food preferences and the behavioral dominances. On the other hand, control treatments were performed using separately two boron compounds added to sucrose solution which was not highly concentrated to avoid constrains due to the viscosity.

Most of the species in the food preference test accepted sugary food; only one species was recorded to visit it less than the protein foods. This result was consistent with the efficacy of control treatments by sugary baits within the rooms. For species that showed good acceptance of sugar solutions in the preference test outdoors, sugar bait control indoors was 100& effective. Conversely, for the only species that foraged significantly less on sugar food, the bait treatment was ineffective. This work reveals the importance of considering the behavior and feeding preferences of the species to be controlled by toxic baits.

## Introduction

Many ant species are serious pests in urban environments. They may cause damage to structures, electronic devices and affect household residents; in addition, many species can sting and/or bite. Presently there is greater awareness of the risks involved in the indiscriminate release of toxicants on the environments we live and work, thus the demand for environmentally friendly control methods is growing. For ant control in urban settings, insecticides have been commonly used as perimeter barrier treatments (Mallis 1969; Ebeling 1978; Hedges 2010; Klotz et al. 2010). However, fast-acting barriers or sprays may result in ants trapped within the building in treatment (Klotz et al. 2008,2010) or in colony budding (Hedges 2010; Buczkowski et al. 2005; Oi 2008), even increasing the foraging activity indoors (Oi et al. 1996). The use of toxic baits is, undoubtedly, the least polluting way to control these insects and it is easy to be commercialized and applied both in homes and buildings. Exterior application of a delayed-action toxicant could result in successful control for indoor ant infestations (Oi et al. 1996).

Ants, as insects in general, have on their cuticle different microorganisms (Beatson 1972; Bueno and Campos-Farinha 1999; Santos et al. 2009), therefore they are frequently believed to be involved in food contamination and mechanical vectoring of disease agents (Beatson 1972; Fowler et al. 1993; Olaya-Masmela et al. 2005; Moreira et al. 2005). In addition, as there are minute species, they can enter places where other, larger insects, cannot (such as cockroaches, flies, mosquitoes, etc.). Moreover, ants travel long distances searching for food, circulating in these trails from several to hundreds of individuals. These characteristics give them the potential capacity to spread pathogens in sensitive places that should be kept aseptic (such as surgical environments, sites for storing sterile instruments or equipment, etc.) and move from hospital areas that may harbor pathogens to patients and between them (Beatson 1972).

Hospitals require careful attention; neonates, infants, elderly or immunosuppressed patients are more sensitive to insect stings and to different pathogens that insects could transport, but also to the pesticides used for their control. Pesticides in sprays, liquids or powders could represent a risk to these patients (Owens 2003). For these reasons, food baits containing a toxicant are recommended to control ants and cockroaches in these sensitive places (Owens 2003; Pampiglione and Velo 2010). This method has many advantages over others: on the one hand, it avoids toxic volatiles and reduces human toxicant exposure, and localization or access to the nest is not necessary as the ants distribute the bait amongst themselves. As social insects, ants live in a colony which presents division of labor among members. The reproductive caste rarely leaves the nest, but a proportion of the sterile workers do in order to forage on different resources. Hence, foragers themselves transport the bait towards the nest, where it is distributed among the remaining colony members. For these insects, the toxicant should have a delayed action, giving them enough time to allow foraging, transport to the nest, sharing therein and recruitment.

There are commercial toxic baits with different toxicants for most urban ants. They are commercialized in different countries as gels or similar vehicles contained in syringes. However, their success is variable (O’Brien and Hooper-Bui 2005; McDonald 2012). This could be due to many reasons that affect the behavior and decision making of the ant individuals during foraging. Foragers modulate their behavior according to the colony needs (Howard and Tschinkel 1980; Josens and Roces 2000; Falibene et al. 2009), the bait properties (Josens et al. 1998; Cassill and Tschinkel 1999; Medan and Josens 2005), the toxicant and its concentration (Hooper-Bui and Rust 2001; O’Brien and Hooper-Bui 2005; Sola et al. 2013), among many other factors. It is known that nectar ingesting ants do not always reach their full crop load and usually leave the food source with partial loads (Josens et al. 1998; Dornhaus et al. 2006). Highly viscous solutions are ingested at a very low rate (Josens et al. 1998; Medan and Josens 2005; O’Brien and Hooper-Bui 2005); ants drink longer and reach smaller loads than for more dilute or less viscous solutions (Josens et al. 1998). Besides, foragers can accept or reject certain baits, recruit other ants or not and intake faster or slower from a certain fluid depending on their motivation to feed (Howard and Tschinkel 1980; Josens and Roces 2000; Falibene et al. 2009; Falibene and Josens 2008; Mailleux et al. 2006). Moreover, behavioral responses to a certain toxic bait are not always the same among different species. For two species with similar feeding habits, like the carpenter ant *Camponotus mus* and the Argentine ant *Linepithema humile* (Newell and Barber 1913; Markin 1970; Hansen and Klotz 2005), boric acid can affect acceptance negatively for the former but not for the latter. On the other hand, borax may cause the inverse situation (Sola et al. 2013). Therefore, all these factors should be considered when developing baits for ant control.

Traditionally, the toxicological effects of pesticides were studied in cases of acute poisoning by accidental exposure to high doses of insecticide, which are well documented (Shannon et al. 2007). However, nowadays there is greater awareness of the risk of long term effects attributable to frequent exposure to doses of low concentrate pesticides (Kapoor et al. 2011; Herin et al. 2011). Also, new studies revealed the close relationship between the chronic health effects that become apparent at a given moment in life and the prolonged or repeated exposure to low doses of pesticides at earlier ages (Winans et al. 2011; Bailey et al. 2011). In this regard, *pediatric* hospitals should consider that their patients are particularly sensitive to pesticides, as they might have an impact on the development of a child and also contribute to the onset of diseases later (Winans et al. 2011). For example, several reports suggest an increase in the incidence of acute lymphoblastic leukemia in children by exposure to pesticides (Leiss and Savitz 1995; Infante-Rivard et al. 1999; Alexander et al. 2001; Thompson et al. 2001; Ma et al. 2002; Menegaux et al. 2006; Pombo-de-Oliveira and Koifman 2006; Rudant et al. 2007). Recently, a study conducted in Australia showed a correlation between the application of pest control treatments in homes and the posterior incidence of the mentioned pathology and also revealed a critical age period for children (2 to 3 years old) (Bailey et al. 2011). The authors argue that this window of greater sensitivity could be related to a greater exposure due to the habits and typical behavior of that age.

Taking all this into account, the pediatric hospital “Hospital de Niños Ricardo Gutiérrez” from Buenos Aires City has generally been trying to avoid the use of pesticide sprays or powders and has only been using commercial baits. Consequently, it has been facing some difficulties to control ant populations within the grounds of the hospital. Owing to this, we were summoned in September 2011. Thus, our challenge was to develop a protocol to control ants in the hospital that would improve the standards with commercial baits. In order to achieve that, we performed two different phases: 1) evaluation of the species present and some aspects of their behavior, and 2) control using baits.

## Material and methods

### The place

The public pediatric hospital in this study occupies 3 hectares in the City of Buenos Aires (34° 35′ 39.20″ S; 58° 24′ 40.76″ W). At the site, there are around 18, 1 and 2 storey buildings interspersed with green spaces with different types of vegetation: trees, grass and ornamental plants. This hospital moved to these grounds in 1896. Several of the present buildings were constructed at that time. The hospital has 300 beds and approximately 2500 medical doctors and nurses work therein; thousands of people circulate there per day.

### Evaluation phase

The objectives of this phase were to evaluate which species were present in green spaces, quantify their abundance, assess their food preferences and determine which were behaviorally dominant.

The individuals collected from manual sampling, pitfall traps and food baits outdoors and also those found indoors were used to make a list of species present in there. The genus abundance was quantified from pitfall traps, and behavioral dominance and food preferences were estimated by means of food baits. Samplings were performed from December 2011 to January 2012, which corresponds to late spring and summer in our latitude.

#### Manual sampling

Six trained students toured the grounds sampling ants in the vegetation, grass, walls, pedestrian paths, and indoors, in order to identify the species and document which of those were associated with buildings. Samples were preserved in ethanol (96&) in bottles labeled with the capture site and date. Manual samplings were performed on two different days between 10 am and 3 pm. The only objective of this sampling was to contribute to the list of species present in the hospital.

#### Pitfall trapping

24 non-baited pitfall traps were placed twice (two weeks apart) in different green spaces of the hospital and left for two days. Each trap consisted of a 200 ml plastic cup buried in the ground and half-filled with alcohol (70&). Pitfalls were left for 48 h, which allowed sampling ants with low activity at the times we did not have access to the hospital. Trapped ants were identified to species or genera with available keys (Kusnezov 1951,1978; MacKay and MacKay 2002; Fernández 2003; Wilson 2003). Relative abundance per genus was measured as the number of ants per genus over the total number of ants collected during the study.

#### Baiting

Food sources were used in order to study the first genus in discovering the resource, the recruitment dynamics and the behavioral dominance among the different genera (Fellers 1987; Savolainen and Vepsäläinen 1988; Andersen 1997; Morrison et al. 2000). Four different types of food were offered simultaneously on “a station” (a square plastic sheet of 20 cm × 20 cm): commercial canned tuna in oil, commercial canned corned meat, honey-water and raw lean beef in similar amounts (ca. 1 g). Within the square sheet, each food was placed in the center of each quarter (i.e. 10 × 10 cm). A total of 32 baits were placed on 8 stations during 70 min. Every 10 min (from **T0** = *time 0 min* to **T7** = *time 70 min*), a photo of each bait was taken (Canon EOS 400D XTi, Canon EF50mm Compact macro lens); recordings and notes about the species visiting the baits were also made in parallel. Later in laboratory, the *number of ants per species* was counted *per bait* from the photos taken, in conjunction with ant samples taken from food bait stations.

Offering 4 alternative types of food per station also allowed us to evaluate food preferences for the species that visited these stations. The distribution of the different species that visited the 4 food baits was compared by means of Chi Squared Homogeneity test. Then, for each species, the number of ants foraging on each food bait was compared by means of Chi Squared Goodness of Fit Test in order to establish the preferences of each species.

### Control phase

#### The toxic baits

The bait consisted of sucrose solution (20& w/w) added with a toxicant offered in plastic tubes (5 ml and 10 mm diameter) with a plug of non-compacted cotton. It is important that the plug of cotton is set lightly so as not to dry on the outside, but does not drip when tilted down. This solution has low viscosity which is preferred for urban ant baits, because it does not present a mechanical difficulty for small species to ingest (Baker et al. 1985 Josens et al. 1998; Silverman and Roulston 2001; O’Brien and Hooper-Bui 2005; Medan and Josens 2005). The toxics used were two boron compounds: *boric acid* and *borax* (sodium borate). These boron toxicants are two of the few allowed by the toxicological service of the hospital, due to their very low toxicity for mammals.

A low concentration of the toxicant (2& w/v) was used to avoid ant rejection, as observed for different species (Klotz et al. 2000; Klotz 2004).

Whenever possible, two baits were offered together: one containing boric acid (BA) and the other, borax (B). This decision was based on previous data on two species which presented the opposite rejection response toward those toxicants (Sola et al. 2013). As the response of the species to be found could not be predicted, offering two alternatives of boron compounds would increase the probability that one of them would be taken to the nest. If a given species rejected a particular boron compound, it would find and feed on the other.

Due to the constant presence of children, tubes had to be located in hidden or hard to reach places so as not to call their attention. Respecting this premise, tubes were placed as close to the ant trails as possible and fixed to the walls with tape (masking tape or duct tape) of a similar color to the background.

**Figure 1:**
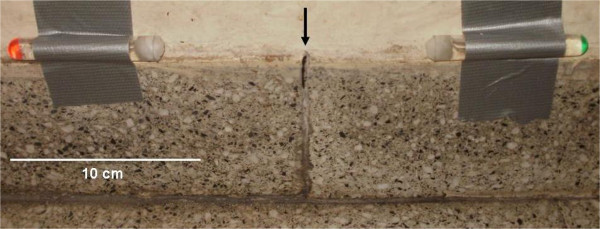
**Bait tubes arrangement.** Sets of two tubes were used: one tube with Boric Acid (BA) and the other with Borax (B). The black arrow indicates the hole that the ants use to exit.

The control phase was performed at two scales: an *extensive outer baiting* for highly infested pavilions, and a *focal*, interior or exterior baiting for particular rooms where staff reported the presence of ants. For the former, sets of two tubes which were 10 ± 5 cm close to each other were placed on the outer walls, (one tube with B and the other with BA Figure 1). The content of the tubes was refilled three times per week when needed, and completely replaced once a week. On those days, ants were sampled to identify the species present in each tube. For the latter, depending on the room and the situation of the child therein, we placed a set of 2 tubes (one with B and the other with BA) or only one tube (with B or BA depending on the species present). Tubes were also controlled three times a week in some rooms and refilled when needed. However, the treatment proved to be effective even in places without performing those refilling tasks, which demanded many man-hours, but placing several tubes at the same time.

#### Highly infested pavilions and Focal infested rooms

Since the beginning of the cooperation with the hospital, its director instructed both the staff and the pest control personnel to notify us if ants were detected in the interior of the buildings.

Our first step in this control phase was to identify the pavilions highly infested with ants. For that, two kinds of recordings were considered: the detection of high ant activity on outer walls and the reports of the hospital staff from the rooms. The ant activity on outer walls was evaluated by means of an outside building visual survey for detection of ant trails by a group of 6 trained students who toured the site.

## Results

### Evaluation phase

Considering all the sampling methods together, 15 species belonging to 12 genera were found (Table 1). Even though 14 species were detected in green areas, most were not associated to building walls. The less common species in the green areas were found only in one sector and in a very low density. The most problematic species was *Nylanderia fulva*; it was abundant in green areas and very frequently associated to buildings and also indoors.

**Table 1:** **List of ant morphospecies detected in the hospital considering every sample method used: pitfall trapping, baits and manual sampling either inside the buildings or outside areas**

Subfamily	Species	Observations
Ponerinae	*Gnamptogenys*	Extremely rare. Individual ants on the grass, no trails were observed.
Formicinae	*Brachymyrmex patagonicus*	Abundant presence. Very small species; usually enters through tiny gaps and cracks in buildings or nests therein.
	*Brachymyrmex australis*	Abundant presence. Very small species; usually enters through tiny gaps and cracks in buildings or nests therein.
	*Nylanderia fulva*	One of the most abundant species. Usually enter and nest in the buildings. Long trails alongside the outer walls of several pavilions.
	*Camponotus sp* (prob. *fuscocinctus*):	Extremely rare. Arboreal, associated with green areas
Myrmicinae	*Pheidole sp1* (cf *P. rosae* (Wilson))	Abundant in green areas. Not found associated to buildings.
	*Pheidole sp2* (cf *P. radoszkowskii*)	Abundant in green areas but rarely found inside buildings.
	*Pheidole acutiloba* (Mackay)	Not abundant in green areas. It was found nesting in one pavilion.
	*Solenopsis albidula*	Very small ants. They were found only on land and in the grass.
	*Acromyrmex lundi*	Black leaf-cutting ants, the biggest in the hospital. Nests are in green areas and also associated to buildings.
	*Strumigenys*	Very rare. Only in grass and trash. Very small ants.
	*Monomorium pharaonis*	Small ants. They were only detected in one building.
Dolichoderinae	*Linepithema micans*	Low abundance. In green areas.
	*Dorymyrmex brunneus*	Low abundance. In green areas.
Pseudomyrmecinae	*Pseudomyrmex phyllophilus*	Low abundance. Arboreal, associated with green areas.

### Relative abundance of genera

A total of 4,767 ants were collected in the 24 pitfall traps placed in the green areas of the hospital. Their genera and, when possible, the species were determined for each individual.

**Figure 2:**
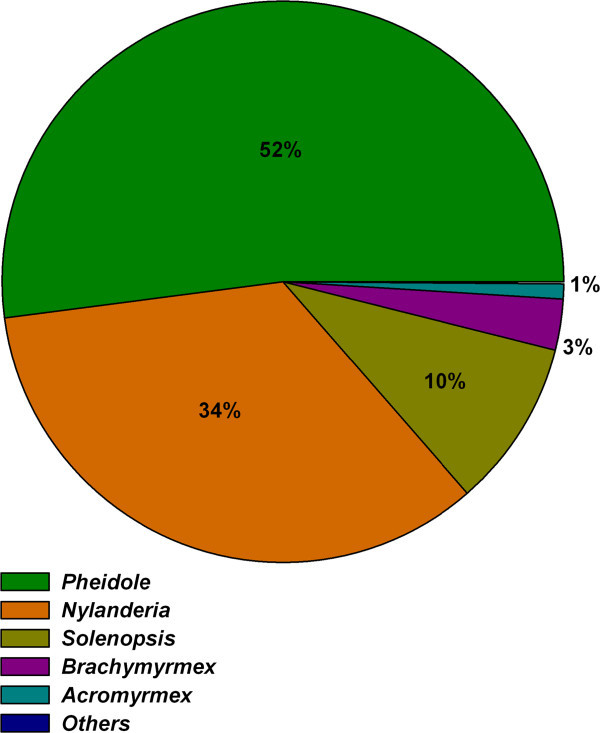
**Relative abundances of ant genera in green areas of the hospital.** Pitfall traps were placed in the green areas of the hospital and left for two days. 4,767 ants were collected and identified.

The most abundant genus was the *Pheidole* (three species) with a 52& of the total abundance, in second place *Nylanderia* (one species) with 34&, followed by *Solenopsis* (one species) with 10&. The rest of the genera represented less than 5&: *Brachymyrmex*, *Acromyrmex*, *Gnamptogenys* and *Strumigenys* (Figure 2).

A more detailed observation of each sampling site revealed that *Pheidole* and *Nylanderia* were found together in each and every site (data not shown). That means they coexisted in all the green places and seemed not to be partitioned across the premises.

### Behavioral dominance

Six ant species visited the stations which offered 4 different food baits each. Table 2 shows the first genera to arrive at each station (at time T0 or T1) and the relative abundance per station at the end of our records (at time T7).

**Table 2:** **Visits and behavioral dominance at the bait stations**

Station	First genera to discover/recruit to baits	Time of ***Nf*** presence***.*** > 3 workers*	Time of Nf recruit. > 20 workers**	Species (&) at T7 (time = 70 m)	
*ST 1*	*Pheidole, Solenopsis*	T5	T5	*N. fulva*	(100&)
*ST 2*	*Pheidole*	T4	T6	*N. fulva*	(97&)
*ST 3*	*Pheidole*	--	--	*P. sp2*	(98&)
*ST 4*	*Pheidole*	T7	T7	*N. fulva*	(43&);
				*B. patagonicus*	(37&);
				*P. sp2*	(15&)
*ST 5*	*Pheidole*	T5	T6	*N. fulva*	*(100&)*
*ST 6*	*Pheidole*	*--*	--	*P. acutiloba*	*(91&)*
*ST 7*	*Pheidole*	*--*	--	*P. sp1*	*(97&)*
*ST 8*	*Pheidole*	T5	--	*N. fulva*	(55&);
				*P. sp2*	(45&)

First genera to arrive at each station (STi), presence of *Nylanderia fulva* as the first time in which 3 (presence) and 20 (recruitment) workers were detected and behavioral dominance showed as the percentage of ant species present at the end of the recording (70 min, T7).

ST: Station; *Nf*: *Nylanderia fulva*; N.: *Nylanderia*; P.: *Pheidole*.

*; ** : First Time (from T0 to T7) at which *N. fulva* had at least 3 (*) or 20 (**) workers at the station.

--: There were no workers or did not reach the indicated number.

**Figure 3:**
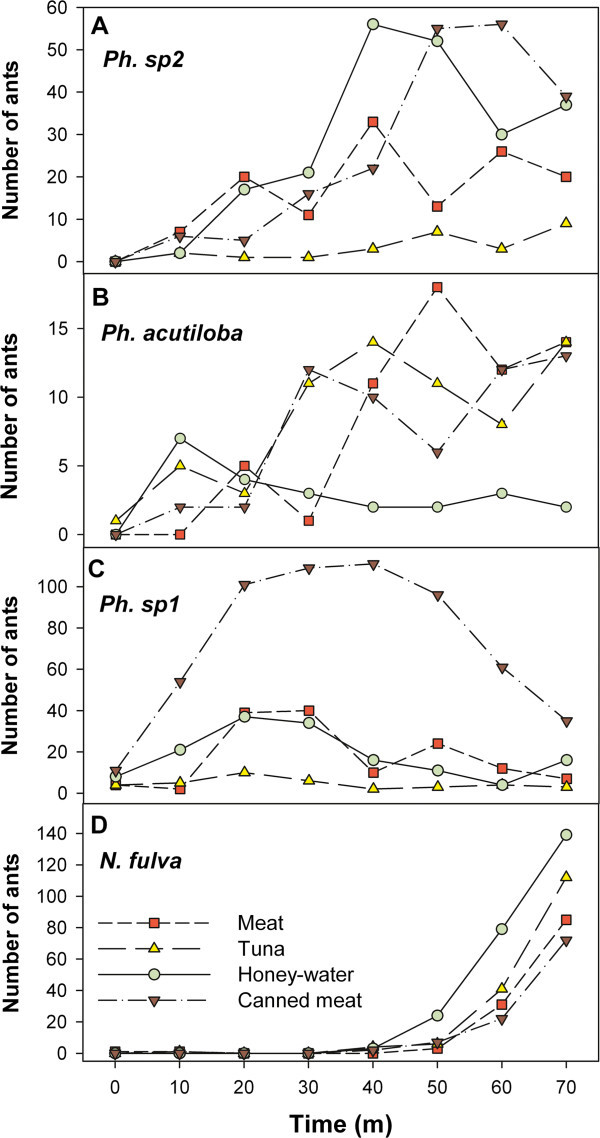
**Temporal dynamics of the species visiting bait stations offered in the green areas of the hospital.** Eight stations were set during 70 minutes, offering meat, oil tuna, canned meat and honey-water. Species and number of ants per species were recorded every 10 minutes. Data showed are the 8 stations pooled for the 4 most numerous species visiting the baits: *Pheidole* sp2 **(A)**, *Pheidole acutiloba* **(B)**, *Pheidole* sp1 **(C)** and *Nylanderia fulva* **(D)**. Note the different scales for each species.

Regarding the temporal dynamics of the species visiting the baits (Figure 3), *Pheidole* sp2 (cf. *P. radoszkowskii*) was the first to arrive at all the stations. Every station was visited by the 3 species of *Pheidole* immediately after they were placed (time 0 or time 1). This probably reflects the fact that *Pheidole* ants are the most abundant in green areas. They could dominate a station if *Nylanderia* did not appear there. *Nylanderia* dominated a station if they found it (see Stations *ST1*, *ST2* and *ST5* in Table 2). When *Nylanderia* encountered the station, they recruited large numbers of individuals and *Pheidole* retreated as *Nylanderia* increased in number (Figure 3).

*Nylanderia* ants discovered 5 out of the 8 stations, mostly, between 40 and 50 minutes after their placement and only then, recruited workers did begin to increase in number and the gradual dropping of *Pheidole* started. In some stations where *Nylanderia* arrived after 40 min, our recordings ended before this process was completed (see Station *ST4* and *ST8* in Table 2).

In conclusion, *Nylanderia* seems to be the behaviorally dominant ant due to their behavior of massive recruitment.

### Food preferences

The different species that visited the baits have similar feeding habits, they are omnivorous and opportunistic. However, some differences were observed in the presence of ants of each genus among the four types of food baits offered (**M**eat, **T**una, **H**oney-water, **C**anned meat).

**Figure 4:**
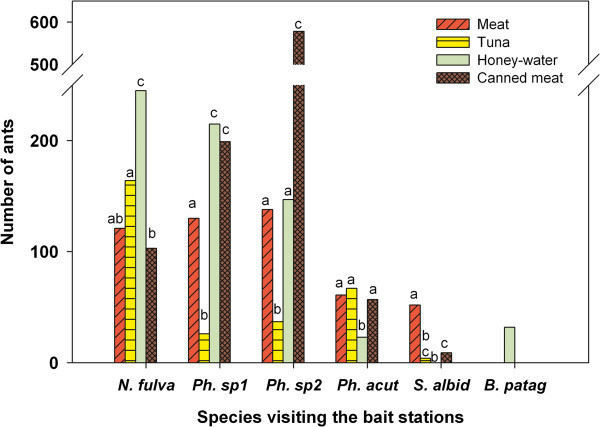
**Preferences of each species among the four food baits offered.** The 8 stations (Figure 3) and their times were pooled. For each species Goodness of fit tests were performed among food types. No letters in common among food types in each species means differences in the preference of worker visits.

The three *Pheidole* species and *N. fulva* were the most abundant species at the baits and they were found in all types of food baits. However, their distribution among baits was not similar (Figures 3 and 4; Homogeneity test for all records pooled: P < 0.001).

Considering each of these four species in particular, none of them visited the four food baits equally. Each species showed significant differences in the number of ants visiting the four food baits; that is at least one food type was either more or less visited than the others (Figure 4; Fit-Goodness Test: P < 0.001 for each species).

Although *Nylanderia* was found in all the foods offered, the sugary bait was the most visited and showed the fastest recruitment; tuna was preferred over the canned meat. In the case of *Pheidole*, the tuna bait was the least visited by the two species of *Pheidole* mostly represented (*P. sp1* and *P. sp2,* cf. *P. rosae* and cf. *P. radoszkowskii*, respectively). *P. sp1* showed a preference for honey-water and canned meat, while *P. sp2* mainly visited canned meat over the other baits. *P. acutiloba* (Mackay et al. 2011) visited the honey-water significantly less compared to the other baits.

*Brachymyrmex* and *Solenopsis* were found visiting the baits in low numbers. *Solenopsis albidula* was found in two stations visiting at least once the Meat, Tuna and Canned meat, but with a clear preference for the meat. *Brachymyrmex patagonicus* was observed in only one honey-water bait.

Even though we only had 8 stations, the patterns of preferences described in the previous lines were consistent. The same pattern was observed when considering all the stations together (Figure 3), only the station where each *Pheidole* species was dominant (Table 2: Station *ST6* for *P. acutiloba*; Station *ST3* for *P. sp1*; Station *ST7* for *P. sp2*), or even considering only the times that each species was dominant in the stations (i.e. “before” *Nylanderia* arrived).

### Control phase

#### Protocol

Many details of the protocol to be developed indoors were determined after trying different alternatives in laboratory and from our previous research. In general terms, the protocol followed some guidelines.

As a general rule to be applied in each case, some individual ants were *sampled* in order to *identify the species* and conserved in alcohol (96&) in flasks labeled with the capture site and date.

Regarding the collocation of the bait tubes, they were placed *horizontally* to ensure that the cotton plug was wet as long as possible. To promote the ants to find the mouth of the tube rapidly, a line of sugar solution was painted by means of a thin brush so that it connected each tube mouth with the ant trail. The procedure described above was followed on every occasion: two tubes close to each other were placed with two boron baits -whenever possible- in hidden places, attached horizontally with tape of the background color, etc. (see Materials and Methods).

#### Highly infested pavilions

The reports received from the hospital staff about ants found in rooms revealed that most of them were located on the ground floor and that several buildings along the premises were highly infested.

Several very active trails in the outer walls were found from the *outside building visual survey*. Particularly, in three buildings where ants walking on the edge of a wall always entered the building, either through a window or through small cracks or holes in the wall.

As a general pattern, there was a correspondence between the buildings with a lot of reports in rooms and buildings with high level of activity outside.

For the severely infested pavilions, our criterion was to set the outside baits all around the buildings, as it is better to attract ants outward rather than inward.

Following the described procedure, firstly we surrounded 3 pavilions (N, P and F) with an extensive outer baiting where children did not circulate or access. That is to say, there were walls with a lot of tubes because children had no access, and sections of walls that had no tubes at all because children were all around. Moldings or baseboards were used to support and fix the tubes as explained above. In Pavilion N, 28 tubes were placed (14 with BA and 14 with B) and for all those tubes and throughout the days recorded, the only species observed was *Nylanderia fulva*. In Pavilion P, 30 tubes were placed (15 BA and 15 B) and the most abundant species was also *N. fulva*. In Pavilion F, 20 tubes were placed (10 BA and 10 B) and the species recorded were *N. fulva*, one species of *Brachymyrmex* and the leaf cutting ant *Acromyrmex lundi*.

As the weather was turning cooler, *Nylanderia* ants were particularly abundant close to the heating-pipe outlets, a fact that is likely to be accentuated during the winter. In early May 2012, the temperature next to these outlets was 30°C (recorded on the wall next to the ant trail with a laser thermometer), while on other walls was about 20°C.

In these pavilions *Nylanderia* commonly presented active trails up to ca. 20 meters long bordering the outer walls. Ants entered and exited from the building through different places, either small holes or the windows. This suggests a high connectivity between colonies with a unicolonial structure, which is common in this species.

The extensive outer baiting lasted a month and was highly effective in reducing the *Nylanderia* infestation indoors in all the pavilions. After this, no more *N. fulva* ants were detected in rooms with outer walls and in the outside for several months.

#### Focal infested rooms

In those focal rooms where ants were detected by the hospital staff, the entrance hole or crack inside the room through which the ants came in was searched. That was an easy task if there was high ant activity. Otherwise, if there were no ants at the moment of this evaluation, small drops of sugar-water were spread on the counters near the corners where the ants had previously been seen. Then, the first ants that appeared were followed to spot the entry. After that, the drops were cleaned.

Then, it was evaluated if the entrance hole was located in walls that limited the building to the outside or in inner walls.

In case of external walls, whenever possible, ant activity on the outside was searched, particularly in the sector that matched the indoor ant detection. If the same type of ant was on both sides of the wall, we assumed they belonged to the same colony. Following the premise that it was better to attract ants outward rather than inward, baits were placed firstly on the outside.

In case of inner walls, whenever possible, ant activity on the other side of the wall was searched. If the same type of ant was also there, then we chose the less sensitive room for bait placement. Thereafter, small cracks and fissures of the more sensitive room were sealed, since the ants left for the baits in the other room. Only in cases when the other side of the wall had no ants or was inaccessible, a bait station in the affected room was set.

We were very careful in placing the baits as close as possible to the hole where the ants came out; otherwise, they could establish trails on the walls or across the countertops, which clearly had to be avoided, especially if patients were children.

After the extensive outer baiting and regarding the isolated reports of ants detected in *rooms* during 2012, we were warned about other 13 rooms affected by ants in different pavilions. The first 11 rooms were treated between May and July 2012 using one or two tubes per room. The tubes were refilled once or twice a week until no more ants were detected. In some cases, ant foraging on the tubes was massive, and during the refilling we could observe that about 10 ml of 2& boron bait was consumed. For the last two rooms (12 and 13), several tubes were set and refilled only once. Table 3 summarizes data on these procedures, the species found and the results for each treatment.

**Table 3:** **Indoor sites treated with sugar toxic baits**

Site	Place details	Species	Nº of tubes: baits applied	Result + / -	Delay
1	Individual Room 4, U5	*N. fulva*	1: BA	+	5
2	Individual Room 2, U5	*N. fulva*	1: BA	+	13
3	Individual Room 3, U5	*N. fulva*	1: BA	+	13
4	Office, U5	*P. sp1*	2: BA, B	+	5
5	Individual Room B, IC and office	*B. patagonicus*	2: BA, B	+	8
6	Individual Room A IC	*B. australis*	2: BA, B	+	8
6	Individual Room A IC	*P. acutiloba*	2: BA, B	-	(+32)
7	Resident physician’s Bathroom, U5	*A. lundi*	2: BA, B	-	(+30)
8	Office, U10	*N. fulva*	2: BA, B	+	9
9	Individual Room 4, U10	*N. fulva*	1: BA	+	21
10	Individual Room 10, U9	*N. fulva*	2: BA, B	+	15
11	Laboratory and office	*M. pharaonis*	4: BA, B	+	7
12	General room and nurse office	*B. patagonicus*	10: BA, B	+	7
13	Laundry room	*B. australis*	6: BA, B	+	14

Keys: N.: *Nylanderia*; P.: *Pheidole*; B.: *Brachymyrmex*; A.: *Acromyrmex*; M.: *Monomorium*; BA: boric acid; B: borax.

Result +: ants were no longer detected. Result -: ants were still detected. Delay: Days until shown results.

The most frequent species indoors were *Nylanderia fulva*, *Brachymyrmex patagonicus and B. australis*. Out of the 13 rooms affected by ants, 6 corresponded to *N. fulva* (46&). The activity of these ants was always particularly intense. The delay or days until non-detection (Delay in Table 3) ranged from 5 to 21 days. Four rooms were affected by *Brachymyrmex* (31&): two by *B. patagonicus* and two by *B. australis*. In all cases the treatment was effective, ants were no longer detected between the first and second week after placing the baits.

One site (11) had very intense and constant activity of the small Pharaoh ants, *Monomorium pharaonis*. This laboratory consisted of 3 rooms, 2 of which were the only places in the hospital where this species was found. Two tubes (1 with BA and 1 with B) were set in each of these two rooms, and a week later no more ants were observed.

*Pheidole sp1* was detected only once indoors (site 4), and this small colony showed no further activity after 5 days of treatment.

We followed up the rooms treated, both by visual assessment and by querying the staff working therein about ant detection. In all the cases mentioned above, no ants were observed in the following seven months. It is worth mentioning that in any case when ants disappeared from one place, they never appeared in a proximal other place, i.e. out of this first control experience, there was no evidence that the baiting provoked the ants to move from one place to another.

One of the two species in which the treatment was not effective was *Acromyrmex lundi* (leaf-cutting ants). They regularly visited the baits during the outer baiting first and then in indoor baiting. However, it is known that this species is not specialized in sugary solutions but forage for fresh plant material they need as substrate for their symbiotic fungus. For this species, there are commercial granulated baits.

The other species that could not be controlled was *P. acutiloba*. This case required special work and attention because two nests were in a very sensitive area. For the first nest detected, the baits were controlled three times per week and the solutions were replaced once a week at least for a month and it still showed signs of activity. Meanwhile, we observed the ants’ behavior. The activity of this colony was always very low: only one to three individuals came out of the mouth of the nest, and walked slowly without straying more than a few centimeters from it. The furthest they were seen was ca. 20 cm from the mouth. The tubes with the sugar baits were initially placed on the molding that formed a curve between the wall and the floor of the room. In one observation, we noticed that some ants had difficulties climbing over this curved area as they slipped down. Consequently, the tubes were placed at the ground level. Despite our efforts, this nest could not be controlled, and maintained a low level of activity, which could be seen not only by the observation of individual ants walking around, but also by construction material frequently deposited in a semicircle around the mouth of the nest.

## Discussion

### Considerations regarding the developed protocol

The protocol showed to be highly effective for the ants that most frequently nested in the hospital buildings (*Nylanderia* and *Brachymyrmex*). Combining two different toxicants –boric acid and borax- has proved to be particularly effective, since in all cases tested, each species visited one toxicant more than the other (data not shown).

The results were evident for patients and hospital staff. In all the sites where control measures were applied, no ants were observed in the following seven months. No further action was taken on those sites, outside or inside. Only until March and April 2013, when summer ended, some of the treated rooms did show ants again. The species were the same as those identified one year earlier; a fact that was recorded for *N. fulva*, *Brachymyrmex* and *Monomorium*. This suggests that the nests were not entirely eliminated, but instead the population dropped. In 2013, the same protocol was applied in the sites where ants resurged and they disappeared once more. Based on this result - and as expected- once or twice annual maintenance applications are recommended at key moments, maybe at the end of summer or early fall. In this temperate climate, where winter has temperatures than can reach zero degrees Celsius (°C), ants tend to congregate in hot spots and heated buildings.

It is often difficult to locate ant nests, but with this protocol it is not necessary. External baiting was effective for the most abundant ant *N. fulva*; as placing baits outside the affected buildings resulted in a dramatic reduction in activity inside. These results coincide with previous studies involving Pharaoh ants (Haack 1991; Oi et al. 1994) and Argentine ants (Forschler and Evans 1994). It was suggested that a toxicant bait formulation could be simply scattered around the exterior periphery of a building, which would be easier to use than placing and removing bait stations (Oi et al. 1996). Even when bait station arrangement and refilling would be harder, certainly the effect of evaporation in hot weather is lower when the solution is within a tube than when scattered. On the other hand, baiting or scattering all along the perimeter is not applicable to sensitive areas such as pediatric hospitals because of the restrictions imposed by the children’s presence. There, bait stations required to be localized in place where children had no access, along the perimeter. Nonetheless, it proved to be effective.

### Considerations regarding the hospital

In relation to the situation of the hospital, there are several issues that hinder ant control within buildings.

The buildings had many small holes that allowed ants enter the walls where they could nest and then exit in the inside. In this sense it was important for us to seal the smaller holes by using silicone and also to work together with the maintenance staff in order to repair deteriorated walls in some sensitive rooms.

The patients’ visitors’ habits seem to be difficult to control. On several occasions we detected ants collecting food in garbage thrown into the bathroom waste basket. Even though visitors are aware that this induces insects to enter the rooms, as indicated by nurses, they state that it is easier for them to do so. It is difficult to change these habits, thus, it should be important to implement some measures in order to increase the visitors’ awareness of the problem or even evaluate an alternative measure so that they can get rid of food wastes in the rooms in a safer way to prevent insect attraction.

### The ants

In this survey on the ant species present in the hospital, we found 7 different ant species indoor and a total of 15 species considering both indoor and green areas. Several studies have demonstrated a great variability of ant assemblage richness and species composition associated with human structures in Brazil, even in hospitals (Fowler et al. 1993; Bueno and Fowler 1994). This is unlike the findings of studies reported from Europe and North America, which have few associated species. It is natural to expect a small number of species in hospitals of temperate climates (Fowler et al. 1993). However, we have found in a hospital of Buenos Aires a similar number of species to several hospitals of Brazil, despite our temperate climate.

It is possible that for some species, the observed activity was low because the ants had different time patterns, being more active at night, dusk or dawn rather than in the middle of the day. However, the pitfall traps could collect and show such an activity as they were left for 48 h. Besides, hospital staff could detect and report ant activity indoors at any time of the day.

*Nylanderia fulva* resulted to be the main species in association with the buildings. In green areas throughout the hospital we saw the coexistence of *N. fulva* with *Pheidole*, *Brachymyrmex* and *Solenopsis*. This coexistence had previously been observed in other urban environments (McDonald 2012). *Nylanderia* accepted all food baits, but the most visited was the sugary bait. This is in accordance with other studies, where sugar based baits were preferred over protein in the laboratory (Cook et al. 2011) and in the field (Lynch et al. 1980). Another study found that *N. fulva* was apparently not attracted to oil based baits (Drees et al. 2010); however, tuna in oil was the second most accepted bait in this study.

*Nylanderia* was typically found in trash cans and restroom drains, but it was also observed in the beds of patients. Oral reports from nurses indicated that if dextrose serum leaked during routine handling, ants would subsequently appear.

A recent study on *N. fulva* (McDonald 2012) mentioned that it is not attracted to most commercial ant baits but one granular product. Some treatments involving insecticides applied to surfaces provided temporary buffer zones around structures, only partially controlling *N. fulva*. Field assessments of the only commercial bait that *N. fulva* found attractive was not effective for control: reduction in the number of ants was low, and after the third or fourth week numbers returned to levels prior to treatment. Although other authors (Zenner-Polania 1990) concluded that effective control of *N. fulva* could not be accomplished through a single control method, we found that our bait and application method were sufficient to control localized populations for up to 7 months. We do not disregard the fact that in temperate climates, harsh winters can work in favor of extending control effectiveness.

*Brachymyrmex* was observed in kitchens, offices, laundry rooms; also in neonatal units. These ants showed an intense activity and quick visits to the baits once placed. *Brachymyrmex patagonicus* was described as an invader ant in the USA, where it nests in natural and disturbed areas, and it is also found in very high numbers in hospitals and other buildings (MacGown et al. 2007). Its diet consists largely of honeydew of different insects, so it is expected to readily recruit to sweet baits. In fact, this was observed in the food bait stations where they were detected only in the sugary bait and also in the effectiveness of the toxic sugar bait used for control. *Brachymyrmex* ants have been found as the most frequent species in some hospitals in Porto Alegre, Brazil (Garcia et al. 2011).

*Monomorium pharaonis*, the Pharaoh ants, were detected only indoors, which coincides with observations for this species which nests primarily indoors in temperate regions (Ebeling 1978). The sugar toxic baits were well accepted and promptly effective. These ants compensate for what they are lacking nutritionally by foraging on either carbohydrates or proteins (Edwards and Abraham 1990). *M. pharaonis* was dominant in several hospitals in Brazil (Fowler et al. 1993; Lise et al. 2006), as well as in temperate climates such as in England (Edwards and Baker 1981). Nevertheless, we only found this species in one laboratory of our hospital in Buenos Aires.

Generally speaking, *Pheidole* is a very diverse genus composed mainly of omnivorous ants. Some groups of species were characterized as specialized, either strict granivores or carnivores (Kusnezov 1951). Carnivores can forage on dead or alive insects but also feed on sweet liquids such as honeydew or extrafloral nectars (Blom and Clark 1980). However, *P. grallipes* that was thought to be exclusively entomophagus, resulted to have a more diversified diet (Clark et al. 1986), and two other *Pheidole* from Argentina also revealed that they were less specialized than expected (Pirk et al. 2009). In a hospital in Brazil, a *Pheidole* ant visited equally sugar and protein baits (Pesquero et al. 2008), which coincides with two species of *Pheidole* found in our study which collected protein and sugar bait as well.

*P. acutiloba* preferred the sugary baits less than the protein baits. Considering that sugar was not a main resource for this ant, in the indoor control situation, after some weeks of no response toward the boron baits, we tried a mixed amino acid-sucrose solution; however, no signs of ants drinking on it were observed either. Therefore, it is possible that this species avoids liquid baits. A study on a laboratory colony of a *Pheidole* species offering simultaneously 4 different vehicles for 30& sucrose bait, suggested a preference for dry vehicles (Lee 2008).

As mentioned above, *P. acutiloba* proved to be the only species among those observed on the food bait stations that visited significantly less the sugary bait than the other foods. This result could explain the low effectiveness of the toxic bait used with this particular species, confirming the need to consider the food preferences of the target species for bait formulations. Eichler (1990) suggested the use of “pre-baits” for *M. pharaonis* in order to determine their preferences; this recommendation is valid for many species, especially for those whose feeding habits are unknown.

In summary, more and more studies conclude that it is necessary to consider the behavior and nutritional preferences of the target species, and also the toxic to be used for the baits when designing control programs (Lee 2008; Sola et al. 2013). For the species where sugary baits did not work, a bait-preference test could indicate the most appropriate bait to use in that particular case. In this sense, we are still working on increasing the acceptance of sugar toxic baits in situations where they are not well accepted.

As mentioned previously, the fact that the species and locations were the same from one year to the next shows that total eradication of the pest ants was not possible in this instance. However, we were able to control the populations of problematic ants eliminating their detection from inside buildings. Based on this, we suggest consistent and regular population monitoring and the use of an integrated management to achieve proper levels of pest control.
